# Multiple epiphyseal dysplasia: A clinical and genetic study of 12 cases in a Swedish 6-generation family

**DOI:** 10.3109/17453670903473032

**Published:** 2009-12-04

**Authors:** Johanna Dahlqvist, Hanna Örlén, Hans Matsson, Niklas Dahl, Torsten LÖnnerholm, Karl-Henrik Gustavson

**Affiliations:** ^1^Department of Genetics and Pathology, the Rudbeck Laboratory, Uppsala University; ^2^Department of Radiology, Uppsala University and University Hospital, Uppsala, Sweden

## Abstract

**Background** Multiple epiphyseal dysplasia (MED) is a common genetically and clinically heterogeneous skeletal dysplasia characterized by early-onset osteoarthritis, mainly in the hip and knee, and mild-to-moderate short stature. Here we report on a 6-generation MED family with 17 affected members.

**Method** The clinical and radiographic data on the 12 affected members still living were scrutinized. A structured inquiry comprising state of health and MED-related symptoms since birth up to the present time and the osteoarthritis outcome (KOOS) questionnaire were sent to all living family members with MED. The 5 known gene loci for autosomal dominant MED were analyzed for linkage, using fluorescence-labeled microsatellite markers. Linkage was ascertained with markers close to the *COL9A2* gene, which was analyzed for mutations by sequencing.

**Results** We identified an exon 3 donor splice mutation in the *COL9A2* gene in all affected family members. Clinical, radiographic, and questionnaire data from affected family members suggested that MED caused by *COL9A2* mutations starts in early childhood with knee pain accompanied by delayed ossification of femoral epiphyses. The disease then either stabilizes during puberty or progresses with additional joints becoming affected; joint surgery might be necessary. The progression of the disease also affects muscles, with increasing atrophy, resulting in muscle fatigue and pain. Muscular atrophy has not been reported earlier in cases with *COL9A2* mutations.

**Interpretation** In a patient with clinically suspected or verified MED, it is important to perform DNA-based analysis to identify a possible disease-causing mutation. This information can be used to carry out genetic risk assessment of other family members and to achieve an early and correct diagnosis in the children.

## Introduction

Multiple epiphyseal dysplasia (MED) is an autosomal dominant skeletal dysplasia that affects approximately 1 in 10,000 individuals. It was first described in 1937, by the Swedish radiologist Ribbing. The predominant features of the disease are delayed and irregular ossification of epiphyses and early onset of osteoarthritis. The MED symptoms appear during early childhood, usually with pain in the knees after exercise. Children who are affected often have difficulty in getting up from the floor and have a waddling gait. They frequently complain of fatigue during long walking. Adult height is usually in the lower range of normal. The limbs are relatively short in comparison to the trunk. Pain and joint deformities progress with age, resulting in early-onset osteoarthritis, particularly of the large weight-bearing joints. The spine is usually normal (Briggs and Chapman 2002, Jakkula et al. 2005, Zanki et al. 2007). The diagnosis of the dominant form of MED is based on physical examination and radiographic findings in the proband and other family members (Lachman et al. 2005).

In many cases, the disease-causing gene mutation is known; mutations in 5 different genes have been identified to cause dominant MED. These genes are *COMP*, which encodes cartilage oligomeric matrix protein; *COL9A1*, *COL9A2*, and *COL9A3*, which code for the 3 alpha chains of cartilage-specific type IX collagen; and *MATN3*, which encodes matrilin-3, a cartilage extracellular matrix protein. *COMP* is mutated in 80% of all MED samples analyzed. In approximately 10–20% of all samples tested, a mutation cannot be identified in any of the 5 known genes mentioned above, suggesting that mutations in other hitherto unidentified genes are also involved in the pathogenesis of dominant MED (Briggs and Chapman 2002, Zanki et al. 2007). There is also a rare autosomal recessive form of MED that is caused by mutations in a sulfate transporter gene *(DTDST).* This form of MED is characterized by malformations of the hands, feet, and knees, with a double-layered patella and scoliosis (Ballhausen et al. 2003).

We present a clinical and genetic study of a 6-generation family with autosomal dominant MED. The individuals affected were of different ages and some of them had had MED symptoms for almost 90 years. The aim of the study was to investigate the symptoms of the affected family members, to analyze the development of the disease over time, and to identify the mutation that causes the disease in this family.

## Material and methods

### Family material and clinical investigations

**Figure 1.**
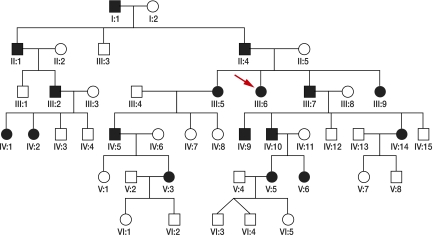
Pedigree of the 6-generation family showing the 17 members affected by multiple epiphyseal dysplasia. Black symbols represent affected individuals. An arrow indicates the proband.

In the present 6-generation MED family, 17 members were or had been affected (Figure 1). The hospital records with clinical and radiographic data concerning the 12 affected members who were still living were scrutinized. 12 cases in 3 generations of the family have been examined earlier clinically and radiographically (Ödman 1959). A structured inquiry covering the state of health and MED-related symptoms from birth up to the present time and also the Swedish version of knee injury and osteoarthritis outcome (KOOS) questionnaire (Roos et al. 2003) were sent to all living family members with MED.

### Genotyping of candidate genes and mutation analysis

Blood samples were collected from 24 family members (12 affected, 9 healthy, and 3 whose disease state was unknown) after obtaining informed consent. Genomic DNA was extracted from peripheral lymphocytes according to standard procedures. The 5 known gene loci *(COMP, MATN3, COL9A1, COL9A2, COL9A3)* for autosomal dominant MED were genotyped using fluorescence-labeled microsatellite markers and 25 ng of DNA per PCR reaction, as described previously (Klar et al. 2004). Genotypes were analyzed with PeakScanner v.1.0 (Applied Biosystems), haplotypes were drawn with Cyrillic v.2.1.3, and 2-point logarithms of odds (LOD) scores were calculated using MLINK (from the LINKAGE package v.5.1) (Lathrop and Laouel 1984). The *COL9A2* gene was analyzed for mutations by DNA sequencing of exon 3 and intron 3, using the BigDye Terminator v3.1 Cycle Sequencing Kit (Invitrogen) and an ABI PRISM 3730 DNA analyser (Applied Biosystems). The DNA sequences obtained were analyzed with Sequencher v.4.1.2 (Gene Codes Corporation, Ann Arbor, MI).

## Results

### Clinical findings

**Figure 2.**
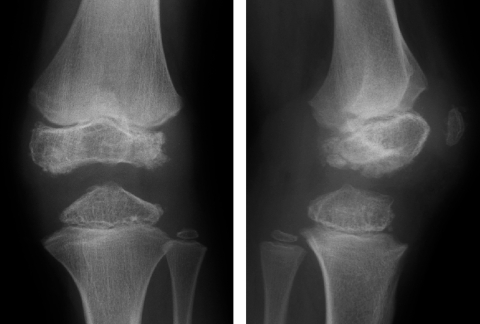
Subject IV:10. Left knee at age 12. The figure shows typical findings with irregular ossification of the epiphyses and delayed ossification of the patella.

**Figure 3.**
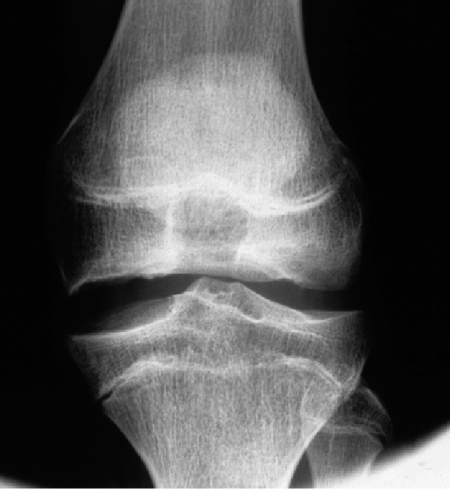
Subject IV:2. Left knee at age 15. The femoral epiphyses are flattened, especially the medial one.

**Figure 4.**
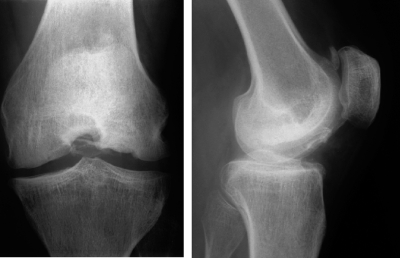
Subject III:6. Left knee at age 40. The femoral condyles are flattened and there is obvious osteoarthritis. A separate bone fragment dorsal to the patella is seen in the lateral view.

The presenting symptoms of the 12 living family members with MED were pain in the knees and fatigue during long walks at 2–7 years of age (Table). The main radiographic findings were delayed ossification of the epiphyses of the hips and knees, irregular bone structures, and irregular jagged contours of the distal femoral epiphyses (Figure 2). Most of the individuals affected had relatively mild manifestations during early adulthood (Figure 3), but at 35–40 years of age many suffered from increasing pain in the knees and hips, resulting in functional disability. Restricted movements of the elbows and knees were common. Degenerative osteoarthritis and sometimes osteochondritis dissecans (Figure 4) was seen on radiographs. A few cases had undergone surgical removal of loose cartilaginous fragments from the knees. In a few cases, total hip replacement was required. After the age of 40 many had developed a scoliosis. All the affected family members had had slim muscles since birth, and after the age of 35–40 slight-to-moderate muscular atrophy was common. In one of the patients, severe muscular atrophy after the age of 80 was seen. The adult heights of the MED patients were in the lower normal range or slightly shorter. The hands and feet were small.

**Table:** Clinical findings in the MED patients

Patient	Present age	Sex	Age at onset of knee pain	Height (cm)	Age at onset of hip pain	Age at surgery
III:2	95	M	5	170	60	93 (THR)
III:6	93	F	4	174	55	65, 76 (THR); 19, 85 (knees)
III:9	86	F	6	160	60	77 (THR); 32, 44 (knees)
IV:1	67	F	7	159	–	50 (elbows)
IV:2	65	F	7	161	–	43 (knees)
IV:5	71	M	2	183	–	–
IV:9	60	M	4	172	–	–
IV:10	59	M	4	170	–	–
IV:14	52	F	4	161	30	36, 42 (knees)
V:3	42	F	5	178	–	–
V:5	31	F	3	153	–	–
V:6	22	F	3	157	–	–

THR: total hip replacement.

The disorder varied strikingly within the family. For example, a 95-year-old male patient (III:2) had a relatively mild form of the disease with only slight pain in the knees during early childhood. He was then free of symptoms until the age of 27, after which he had a few episodes of pain in the knees caused by osteochondritis dissecans. No surgery was needed. By the age of 60 he suffered from slight pain in the hips, and this pain began to increase at the age of 88, requiring bilateral hip replacement when he was 93. In contrast, a 93-year-old female patient (III:6) had a severe form of MED. From the age of 4, she had pain in the knees when weight bearing and difficulty in walking. At the age of 14 she began to suffer from dull ache in the knees. Knee surgery with removal of loose cartilaginous fragments was performed when she was 19 years old, and again at the age of 85. After the age of 35 she had increasing, disabling pain in several joints and in muscles. She had total hip replacements at the age of 65 and at 76. At the age of 85 severe muscular atrophy developed, with motor disability, and she became confined to a wheelchair.

The 8 females affected had a more severe form of MED than the 4 males, and together they had undergone 11 orthopedic operations. Only 1 of the 4 males needed orthopedic surgery. All the adult family members affected had a university education and full-time employment.

### Linkage and mutation analysis

Haplotype construction and linkage analysis of the 5 gene loci for autosomal dominant MED was performed with DNA from the 24 family members. Linkage was excluded for the *COMP, MATN3, COL9A1*, and *COL9A3* loci. A positive linkage to the disease was obtained with marker D1S1598 close to the *COL9A2* gene (maximum LOD score of 2.7). The *COL9A2* gene was analyzed for mutations by sequencing of exon 3 and intron 3—the part of the gene where mutations have been identified in previous MED patients (Muragaki et al. 1996, Holden et al. 1999, Fiedler et al. 2002). A single nucleotide alteration, a T to C transition (IVS3DS [+2] T>C), was identified in the exon 3/intron 3 donor splice site in a heterozygous state in all 12 affected individuals from whom DNA was available. Analysis of the 9 healthy family members and of 3 individuals whose disease state was unknown confirmed that they did not carry the mutation.

## Discussion

Our study of a 6-generation MED family is unique in that the affected family members were followed clinically from infancy to old age—the oldest up to the age of 95. Our findings show that MED caused by this particular *COL9A2* mutation generally starts in early childhood with knee pain, which is accompanied by delayed ossification of femoral epiphyses. The disease then either stabilizes during puberty, with occasional knee pain, or progresses. Frequently, additional joints such as the elbows, hands, and hips then become affected and joint surgery may be necessary. Progression of the disease also affects muscles, with increasing atrophy, resulting in muscle fatigue and pain.

All of the affected family members in our study were found to carry an identical mutation at the donor splice site of exon 3/intron 3 of the *COL9A2* gene, which was dominantly inherited in this 6-generation family. There is a 1 in 2 (or 50%) chance that each child of an affected parent will inherit the mutation and thus be affected with the disorder. The exons of the *COL9A2* gene are translated into protein (a subunit of collagen IX), while the introns of the gene are removed and not translated. When the mutation is found at the donor splice site, it means that it is located right at the site of the cutoff between the exon and intron. Muragaki et al. (1996) previously identified the mutation (IVS3DS [+2] T>C) in one family, and concluded that its effect is that exon 3 is not translated at all, resulting in a loss of 12 amino acids in the protein product. Less than 5% of the mutations that are known to cause dominant MED are located in the *COL9A2*gene; this type of MED is defined as EDM2 (Briggs and Chapman 2002, Jakkula et al. 2005). The EDM2 mutations are all located at different positions within the donor splice site of intron 3 and result in an in-frame deletion of 12 amino acids from the COL3 domain of the collagen chain (Holden et al. 1999, Spayde et al. 2000, Fiedler et al. 2002, Nakashima et al. 2005, Takahashi et al. 2006). The COL3 domain has proven to be essential for the binding between collagen IX and MATN3, an extracellular matrix protein in cartilage (Fresquet et al. 2007). Together with *COL9A1* and *COL9A3*, *COL9A2* forms the collagen IX heterotrimer. Previous studies suggest that collagen IX functions as a bridge between collagen fibrils and matrix macromolecules in the joint cartilage (Olsen 1997).

In addition to the genetic heterogeneity in MED, there is a marked intrafamilial variability in the clinical phenotype, suggesting that environmental and/or modifying genetic factors also play a role (Chapman et al. 2003). Despite intrafamilial differences in severity of the phenotype, all reported cases of EDM2 in addition to our cases have shared the features of more severe involvement of the knees and relative sparing of the hips (Barrie et al.1958, van Mourik et al. 1998, Holden et al. 1999, Fiedler et al. 2002). A few cases with rare additional symptoms, such as double-layered patellae (Nakashima et al. 2005) and ulnar club hands (Takahashi et al. 2006), have been reported. Muscular atrophy, which was common in our family, has not been reported earlier in cases of EDM2. Muscular atrophy has been reported in families with mutated *COMP* and *COL9A3* genes (BÖnnemann at al. 2000, Jakkula et al. 2003).

In a patient with MED, it is important to carry out a DNA-based analysis to identify a possible disease-causing mutation. This information can be used in genetic risk assessment of other family members and to achieve an early and correct diagnosis in children in the family with suspected MED. The risk for each child of being an affected person is 1 in 2, whereas the risk for the offspring of an unaffected person is insignificant. There is as yet no curative treatment for the disease, but much can be done to alleviate the symptoms, counteract deformities, and compensate for functional limitations. Families that have children with MED need to be in early contact with a pediatric orthopedic surgeon; operations may be necessary to minimize joint deformation and to preserve mobility. The families should also be connected to a center for pediatric and youth rehabilitation at an early stage. The occupational therapist can work out techniques to help the child to manage everyday activities at school and at home. The physiotherapist is responsible for evaluation, treatment, and programs for motor training. The various therapeutic components should be co-ordinated to achieve the best possible results. Adults with MED need to continue to interact with an orthopedic surgeon, an occupational therapist, and a physiotherapist, and sometimes with a rehabilitation center for adults. Joint replacement may become unavoidable with age.
